# Immunosensors and Immunoassays to Detect *Francisella tularensis* and Diagnose Tularemia

**DOI:** 10.3390/bios16030158

**Published:** 2026-03-13

**Authors:** Miroslav Pohanka

**Affiliations:** Military Faculty of Medicine, University of Defense, Trebesska 1575, CZ-50001 Hradec Kralove, Czech Republic; miroslav.pohanka@gmail.com or miroslav.pohanka@unob.cz

**Keywords:** biodefense, biosensor, *Francisella tularensis*, handheld assay, immunoassay, monoclonal antibody, point-of-care test, tularemia

## Abstract

*Francisella tularensis*, the causative agent of tularemia, is a highly infectious Category A biothreat agent characterized by an exceptionally low infectious dose and diverse transmission routes. Due to the pathogen’s fastidious growth requirements and the high risk of laboratory-acquired infections, traditional cultivation methods are often protracted and hazardous. Consequently, the development of rapid and sensitive diagnostic tools is paramount. This manuscript provides a comprehensive overview of the current landscape of immunoassays, with a specific focus on the evolution from standard laboratory techniques to advanced biosensors. We detail the critical phases of antigen preparation, including high-pressure homogenization and sonication, and the generation of high-affinity polyclonal and monoclonal antibodies. Furthermore, we evaluate the implementation of novel biosensor-like devices, such as electrochemiluminescence and Surface-Enhanced Raman Scattering platforms, designed for point-of-care and field-ready scenarios. By synthesizing recent advancements in nanomaterial-enhanced recognition and microfluidic integration, this review emphasizes the pivotal role of these technologies in achieving early detection and mitigating the impact of both natural outbreaks and potential deliberate misuse of *F. tularensis*.

## 1. Introduction

*Francisella tularensis* represents one of the most formidable microorganisms in the field of biological threat agents. This Gram-negative, facultative intracellular coccobacillus is the etiological agent of tularemia, a severe zoonotic disease. The biological significance of *F. tularensis* is underscored by its extreme infectivity; the inhalation of as few as 10 to 50 viable cells can precipitate systemic, often fatal, disease in humans. This high virulence, combined with its environmental stability and potential for aerosolization, has led the Centers for Disease Control and Prevention (CDC) to classify it as a Category A priority pathogen [1,2].

The clinical diagnosis of tularemia remains challenging due to its heterogeneous manifestations, ranging from localized ulceroglandular forms to life-threatening pneumonic infections. Traditional diagnostic methods, primarily culture-based isolation, are hampered by the bacterium’s nutritional fastidiousness, requiring specialized cysteine-enriched media, and a slow growth rate that delays therapeutic intervention [3,4]. Furthermore, handling live cultures necessitates stringent Biosafety Level 3 (BSL-3) containment to prevent laboratory-acquired infections. In this context, immunoassays have emerged as indispensable tools for both clinical diagnosis and environmental surveillance. Unlike molecular techniques that may be limited to the transient bacteremia phase, immunoassays can detect both the pathogen (antigen capture) and the host’s immune response (serology), providing a broader diagnostic window [4,5].

The advent of biosensors and biosensor-like devices has further revolutionized this field by transitioning diagnostics from centralized laboratories to point-of-care and field conditions including the assay and diagnosis of biological warfare agents [6,7,8]. These devices offer the advantages of portability, rapid turnaround times, and simplified operation, which are critical in outbreak scenarios or suspected biothreat events. Modern platforms leverage nanomaterials and innovative transducers to achieve sensitivities that rival or exceed traditional Enzyme-Linked Immunosorbent Assay (ELISA) formats [9].

The aim of this manuscript is to provide a systematic summary of the facts and developments in knowledge regarding immunoassays for *F. tularensis*. Specifically, we focus on the transition toward biosensors and biosensor-like devices, detailing the underlying technologies, antigen–antibody interactions, and the practical implementation of these tools in diverse diagnostic scenarios.

## 2. *Francisella tularensis*: Biological Characteristics and Taxonomic Classification

*F. tularensis* represents one of the most significant microorganisms within the field of biological threat agents. This Gram-negative, non-spore-forming, and non-motile coccobacillus, belonging to the family *Francisellaceae*, is characterized by its minute dimensions (approximately 0.2–0.7 μm) and pronounced pleomorphism, which facilitates efficient penetration into host tissues. As an obligately aerobic and facultatively intracellular pathogen, *F. tularensis* has evolved sophisticated adaptive mechanisms to persist across diverse ecosystems, ranging from soil substrates and aquatic reservoirs (often in association with amoebae) to the intracellular environment of macrophages in higher vertebrates [10,11,12].

**Figure 1:**
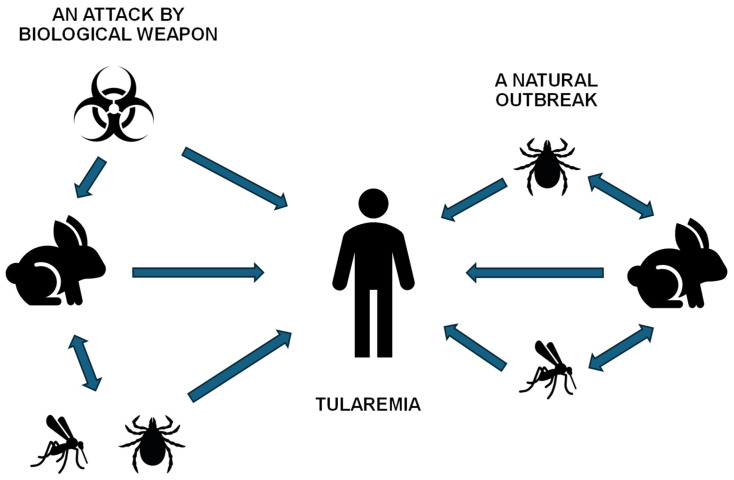
The multifaceted transmission dynamics and ecological life cycle of *F. tularensis*.

The biological uniqueness of this microorganism lies primarily in its exceptionally low infectious dose; theoretically, a mere 10 to 50 viable cells are sufficient to induce systemic disease in a susceptible host, particularly via the inhalation route [13]. This high infectivity, combined with its capacity for zoonotic transmission, classifies *F. tularensis* as a Category A priority pathogen according to the CDC [14]. In its natural ecology, the life cycle of *F. tularensis* is maintained through complex enzootic cycles involving diverse vertebrate hosts, most notably lagomorphs such as hares and various small rodents [15,16,17]. Transmission between these reservoirs and to incidental hosts is frequently mediated by hematophagous arthropod vectors, including hard ticks, deer flies, and mosquitoes, though the specific vector varies significantly by geographical region. In certain regions, an aquatic cycle is also prominent, where the bacterium persists in water and biofilms, infecting semi-aquatic rodents and mosquitoes. Beyond these environmental pathways, the pathogen’s potential for aerosolization presents a severe risk for human infection through deliberate exposure in the context of a biological weapon attack. These multifaceted transmission pathways, encompassing both natural environmental cycles and anthropogenic risks, are systematically depicted in Figure 1.

The taxonomic landscape of the genus *Francisella* has undergone significant revision in recent decades due to advancements in whole-genome sequencing. The primary species, *F. tularensis*, is divided into three primary subspecies—*tularensis*, *holarctica*, and *mediasiatica*, each exhibiting distinct virulence profiles and geographic distributions [18,19,20].

Subsp. *tularensis* (Type A): This is the most virulent subspecies and is endemic to North America. It is responsible for the most severe, often lethal, clinical forms of tularemia [21,22].

Subsp. *holarctica* (Type B): This subspecies exhibits a broader distribution across the Northern Hemisphere, including Europe and Asia. Although it remains highly infectious and spreads rapidly as a zoonosis, its clinical progression is generally less aggressive compared to Type A [23,24,25].

Subsp. *mediasiatica*: Predominantly located in Central Asia, the ecological niche and pathogenic potential of these subspecies in humans remain subjects of ongoing research, though its virulence is considered lower than that of the subspecies *tularensis* and *holarctica* [26,27,28].

Furthermore, the closely related and rare species *F. novicida* is frequently employed in research as a safer surrogate model organism. While it maintains the core mechanisms of intracellular survival, it exhibits low virulence in immunocompetent humans [29,30,31,32]. It should be noted, however, that taxonomic debate persists regarding its status. Some authors propose its classification as a subspecies, *F. tularensis* subsp. *novicida*, due to high genomic similarity with the more virulent subspecies. In contrast, other researchers prefer to maintain the species-level designation *F. novicida* to avoid clinical confusion with the Tier 1 select agent and to respect the significant differences in biosafety requirements and regulatory oversight [33,34,35]. The mentioned subspecies are summarized in Table 1.

The laboratory cultivation of *F. tularensis* presents a significant challenge for microbiologists due to its pronounced nutritional fastidiousness. The bacterium lacks several metabolic pathways required for the synthesis of essential amino acids and, consequently, necessitates specialized media heavily supplemented with sulfhydryl groups, particularly cysteine. The standard diagnostic substrate employed is Cysteine Heart Agar (CHA), typically enriched with blood or egg yolk [36,37]. Growth is characteristically slow, with minute, opalescent colonies typically forming after 48 to 72 h of incubation at temperatures between 35 and 37 °C. Given the high risk of laboratory-acquired infections via infectious aerosols, all manipulation of live cultures is strictly confined to BSL-3 facilities for fully infectious strains, such as those belonging to subsp. *tularensis*. Traditional biochemical identification is further complicated by the pathogen’s weak fermentative activity; thus, modern diagnostics favor molecular techniques, specifically polymerase chain reaction (PCR) targeting signature genes such as FopA or Tul4 [38].

The molecular pathogenesis of *F. tularensis* is inextricably linked to its capacity to subvert the host’s innate immune response. A critical virulence factor is the presence of a polysaccharide capsule, which serves to mask the bacterium from complement components and inhibit opsonization. Upon phagocytosis by macrophages, the bacterium suppresses the induction of the oxidative burst and actively blocks the maturation of the phagosome by inhibiting phagosome–lysosome fusion [39,40]. Utilizing effector proteins encoded within the *Francisella* Pathogenicity Island (FPI), the pathogen disrupts the phagosomal membrane and escapes into the cytosol, where rapid intracellular replication occurs [41,42,43]. A notable feature is the unique structure of its lipopolysaccharide (LPS), which exhibits exceptionally low endotoxic activity. This is due to its inability to effectively bind and activate host toll-like receptor 4 (TLR4), a stark contrast to most other Gram-negative pathogens [44,45,46]. This state of “immunological invisibility” allows the bacterium to proliferate unchecked during the initial phases of infection without triggering an early inflammatory response.

The clinical manifestations of tularemia are highly heterogeneous and are directly contingent upon the pathogen’s portal of entry and its specific virulence. The most prevalent manifestation is ulceroglandular tularemia, which follows cutaneous inoculation (for instance, via the bite of an infected tick, or through direct contact/animal bites); this form is characterized by a necrotic ulcer at the site of inoculation accompanied by painful regional lymphadenopathy (buboes) [47,48]. Bacterial entry via the conjunctiva results in the development of the oculoglandular form, whereas the ingestion of contaminated water or food leads to the oropharyngeal form [49,50,51].

The most severe clinical presentation, however, is pneumonic tularemia, which arises from the inhalation of infectious aerosols [52,53]. This form is marked by rapid progression toward hemorrhagic pneumonia and septic shock; in the absence of appropriate antimicrobial therapy, mortality rates for Type A strains can reach 30–60% [54]. Finally, typhoidal tularemia represents a systemic manifestation lacking a discernible primary lesion, typically presenting with high-grade fever and multi-organ failure [55,56].

The epidemiology of tularemia reflects its status as a classical zoonosis characterized by a complex transmission cycle. Natural reservoirs primarily comprise small mammals, specifically small rodents (voles, mice) and lagomorphs (hares and rabbits), in which the infection often manifests as devastating epizootics [57,58]. Human transmission is mediated either through direct contact with the tissues or bodily fluids of infected animals (e.g., during skinning or dressing of game) or indirectly via hematophagous vectors. Key vectors include ticks of the genera Dermacentor and Ixodes, as well as certain species of deer flies and mosquitoes, the latter being particularly significant in high-latitude regions [59,60,61].

Furthermore, the environmental transmission route plays a critical role in the pathogen’s ecology; the contamination of aquatic reservoirs by rodent excreta can trigger extensive water-borne outbreaks. Historically, *F. tularensis* was first described in 1912 by George McCoy in Tulare County, California, the location from which the disease derives its name. However, its extreme infectivity and potential for weaponization led to intensive characterization within state-level biological warfare programs during the Cold War. This legacy of potential misuse necessitates the stringent international oversight and rigorous biosafety protocols governing the handling of this agent today [62,63,64].

The therapeutic management of tularemia necessitates the prompt administration of specific antimicrobial agents, as the bacterium exhibits intrinsic resistance to beta-lactams. This resistance is mediated by the production of specific beta-lactamases and structural alterations in penicillin-binding proteins (PBPs) [65,66]. Aminoglycosides, specifically streptomycin and gentamicin, remain the definitive drugs of choice due to their potent bactericidal activity [67,68,69]. In less severe clinical presentations or within outpatient settings, fluoroquinolones (e.g., ciprofloxacin) or tetracyclines (e.g., doxycycline) may be employed; however, these bacteriostatic or secondary bactericidal agents are associated with a higher incidence of relapse if the duration of therapy is insufficient [70,71,72].

Disease prevention is primarily based on the education of high-risk cohorts—such as hunters and agricultural workers—alongside the use of insect repellents and personal protective equipment. Although a Live Attenuated Vaccine (LVS) exists, its clinical application remains globally restricted, and no standardized pharmaceutical formulation is currently available for widespread use. Consequently, contemporary research is focused on the development of advanced recombinant and subunit vaccines designed to confer stable protective immunity without the associated risks of residual virulence [73,74,75].

## 3. Important Model Strains

### 3.1. Live Vaccine Strain

The Live Vaccine Strain (LVS) constitutes a unique, artificially attenuated lineage of the bacterium *F. tularensis*, which was developed for the purpose of targeted immunization of high-risk populations [76,77]. The historical origins of this strain trace back to the 1950s in the former Soviet Union, where primary development was conducted at institutes specializing in epidemiology and microbiology, specifically the Gamaleya Institute in Moscow. At this institution, researchers employed the method of serial passage of a virulent *F. tularensis* subsp. *holarctica* isolate on artificial culture media. Through this process, they progressively eliminated the pathogen’s capacity to induce clinical disease while successfully preserving its antigenic properties. In the 1960s, this biological material was transferred to the United States, where further selection and stabilization were performed at the Fort Detrick military laboratories, resulting in the standardized form utilized in contemporary scientific research.

From a microbiological perspective, this organism is a Gram-negative coccobacillus that exhibits pronounced pleomorphism. A defining characteristic of the LVS is its targeted attenuation, which results from specific genetic deletions and mutations. Contemporary genomic analyses have revealed that this weakening is not caused by a single modification, but rather by a combination of defects in genes responsible for the biosynthesis of surface structures and metabolic pathways; these alterations prevent the bacterium from effectively suppressing the host’s immune response [78,79]. Although the strain requires cysteine-enriched media for growth, such as cysteine heart agar, its laboratory cultivation remains relatively stable, if emphasis is placed on maintaining the so-called “blue variant” of the colonies. This specific phenotype is the primary carrier of the required immunogenicity, whereas the transition to the “grey” variant often correlates with a loss of protective efficacy.

The immunological mechanism of action of LVS involves simulating the natural infection process, albeit through a controlled progression. Following administration, activation occurs primarily within the cell-mediated component of the immune system, in which CD4+ and CD8+ T-lymphocytes play a pivotal role [80,81]. These cells, alongside the production of pro-inflammatory cytokines such as interferon-gamma (IFN-γ), prime the host organism to recognize and eliminate the pathogen sequestered within macrophages. Although vaccination also induces the synthesis of specific antibodies, this robust cellular immune response is considered the primary pillar of protection conferred by LVS against subsequent exposure to virulent wild-type strains.

Currently, the LVS serves an indispensable function primarily within the field of biomedical research. Given that it is permissible for use under BSL-2 containment, it functions as a surrogate model for investigating the molecular pathogenesis of tularemia while mitigating extreme occupational hazards to laboratory personnel. Notwithstanding its efficacy, the strain exhibits significant limitations, most notably its failure to confer complete protective immunity against hypervirulent Type A strains following inhalational challenge. Furthermore, the precise molecular basis of its attenuation remained insufficiently characterized for an extended period. Consequently, its application in human medicine is largely restricted to specific investigational protocols, and ongoing research is directed toward the development of next-generation, well-defined vaccine candidates with superior safety profiles and enhanced protective breadth.

### 3.2. FSC200

The FSC200 strain represents a modern scientific counterpart to the legacy LVS and is regarded in contemporary microbiology as a fundamental model organism for the study of *Francisella tularensis* subsp. *holarctica*. Unlike the LVS, which underwent decades of non-standardized serial passage resulting in ambiguous genetic alterations, FSC200 was selected and characterized as a highly virulent, genetically stable, and well-defined representative of European Type B lineages. This isolate originated in Scandinavia, specifically Sweden, where it was recovered from an infected hare. It has since become the reference standard for European laboratories due to its ability to faithfully replicate the phenotypic and genotypic characteristics of wild-type strains circulating among rodent and lagomorph populations. Furthermore, its genome has been extensively characterized in subsequent isolates obtained from clinical cases in humans [82].

From a genomic perspective, FSC200 is highly valued for its genetic integrity, as its sequence lacks the extensive, uncharacterized deletions that attenuate the LVS. This genomic completeness renders it an ideal “parental strain” for modern reverse genetics methodologies. In research settings, it is utilized to generate targeted mutants by selectively deleting specific genes from its comprehensive genetic repertoire, such as those involved in purine metabolism or encoding specific virulence factors [83]. This process facilitates the development of novel, defined vaccine candidates with precisely mapped DNA modifications, representing a fundamental shift away from the stochastic attenuation process that produced LVS.

In a laboratory setting, FSC200 is classified as a pathogen requiring BSL-2 containment. Its virulence in susceptible animal models, such as murine models, is exceptionally high: an extremely low infectious dose (frequently fewer than ten colony-forming units) is sufficient to induce lethal disease. This high degree of pathogenicity enables researchers to meticulously monitor the systemic dissemination of the bacterium, its colonization of the liver and spleen, and the efficacy with which it antagonizes the host immune response during the nascent stages of infection. Comparative analyses between FSC200 and its derived mutants thus yield the most precise data regarding the specific molecular mechanisms that establish *Francisella* as a successful intracellular parasite. A relative disadvantage of FSC200 compared to LVS is the near-absolute mortality rate in laboratory animals, which may limit the window for longitudinal observation of chronic pathological alterations.

The current significance of the FSC200 strain is underscored by its role in the development of next-generation live-attenuated vaccines [84]. While LVS is regarded in many respects as obsolete due to its poorly defined lineage, FSC200 serves as a “clean slate” for the engineering of vaccines that are not only safe but also sufficiently immunogenic to confer protection against the most hazardous Type A (subsp. *tularensis*) strains. Consequently, this strain bridges the gap between European epidemiological history and the most advanced methodologies in genetic engineering.

### 3.3. Schu S4

The Schu S4 strain (frequently abbreviated as Schu) represents the pinnacle of virulence in microbiology and biodefense research and is regarded as the prototypical strain of *Francisella tularensis* subsp. *tularensis* (Type A1). Its history is inextricably linked to American military research; it was originally isolated in 1941 from a lesion on a patient named Charles Schuria, who presented with a characteristic form of the disease [85]. Since its isolation, it has become the most extensively studied strain globally for elucidating the extreme pathogenicity of this microorganism. Its complete genome, sequenced at the turn of the 21st century, serves as the fundamental reference map for all comparative genomic analyses within the genus *Francisella*.

The biological hazard posed by the Schu S4 strain is defined by its capacity to induce fatal infection in both humans and animals following exposure to a negligible quantity of bacteria; in cases of inhalation, the infectious dose is estimated to be in the single digits. At the molecular level, this strain possesses a highly efficient arsenal of virulence factors, primarily encoded within the FPI [86,87,88,89]. These factors enable Schu S4, upon entering a host cell (specifically a macrophage), to rapidly escape the phagosome into the cytosol, where it undergoes unchecked replication while actively suppressing host immune signaling pathways that would otherwise trigger a pro-inflammatory response. This capacity for “stealth” dissemination is significantly more advanced in Schu S4 than in the previously mentioned subsp. *holarctica* strains.

In laboratory practice, manipulation of the Schu S4 strain necessitates the most stringent BSL-3 containment protocols (and in certain contexts, BSL-4). Its phenotype is characterized by rapid growth on enriched media and high biochemical activity, which distinguishes it from attenuated or less virulent variants. Like the LVS, which is attenuated for prophylactic use, or the FSC200 strain, which represents the European Type B lineage, Schu S4 is frequently used as a outcome for vaccine development [90,91,92]. In this capacity, it is deployed during the terminal phases of vaccine and therapeutic testing to verify whether a candidate agent can withstand a challenge from the most aggressive known representative of the species. A distinct disadvantage of Schu S4, like FSC200, is its near-absolute lethality in laboratory animals, which complicates the study of chronic pathological processes or the isolation of convalescent antibodies.

### 3.4. Comparative Analysis of the Schu S4, FSC200, and LVSs

The comparative analysis of Schu S4, FSC200, and LVSs reveals profound phylogenetic and functional discrepancies that determine their respective pathophysiological potentials [93]. The survey of comparative analysis is shown in Table 2. The central determinant of virulence is the *Francisella* Pathogenicity Island (FPI), which encodes a Type VI Secretion System (T6SS) [94,95,96]. While the North American prototype Schu S4 (subsp. *tularensis*) possesses two intact copies of the FPI with a fully functional pdpD gene, the European isolate FSC200 (subsp. *holarctica*) exhibits the natural variability at this locus characteristic of Type B lineages yet retains the capacity for efficient phagosomal escape. In stark contrast, the LVS harbors critical deletions within the FPI region, which radically impairs its intracellular manipulation capabilities and degrades its secretory apparatus to a non-functional level [97,98]. This genetic lesion is the primary pillar of attenuation in LVS; without a robust T6SS, the bacterium fails to timely disrupt the phagocytic vacuole membrane and succumbs to lysosomal degradation before initiating massive cytosolic replication.

Further significant differences reside in metabolic competence and iron homeostasis, mediated by the fupA/B locus [99,100,101]. Strains Schu S4 and FSC200 utilize a high-affinity transporter encoded by the fupA gene, which is critical for survival within the nutrient-restricted environment of the macrophage. In the LVS, however, fusion and partial deletion of these genes have occurred, leading to a pronounced deficit in iron acquisition and a subsequent increase in generation time in vivo. Additionally, the LVS exhibits several secondary mutations in genes associated with nucleotide biosynthesis (e.g., guaA/B) and stress response regulators [98]. These defects, accumulated over decades of in vitro serial passage, result in the inability of LVS to effectively mask its surface structures (LPS and capsule). Consequently, LVS is readily recognized by innate immune receptors, a sharp contrast to the “immunologically silent” profiles of the Schu S4 and FSC200 strains.

The implementation of specific biocontainment protocols for *F. tularensis* is not globally standardized; rather, the mandated biosafety level for identical strains often fluctuates depending on the geographical region and the governing regulatory body of the country where the laboratory is situated. While a strain may be classified as a high-containment pathogen in one jurisdiction, it may be subject to different oversight elsewhere based on local risk assessments and public health history.

Within the European Union Regulatory Framework, the primary legal foundation is Directive 2000/54/EC, which establishes a categorized list of biological agents and their corresponding risk groups to ensure the protection of laboratory personnel. This directive provides a baseline for containment but allows individual member states, such as Sweden or the Czech Republic, to adapt these measures for specific isolates like FSC200, which is frequently handled under BSL-2 conditions in those nations. Conversely, for United States laboratories, the operational standards are dictated by the CDC/NIH Biosafety in Microbiological and Biomedical Laboratories (BMBL). This framework, in conjunction with the federal Select Agent Regulations (42 CFR Part 73), generally mandates BSL-3 oversight for most *F. tularensis* subspecies, while providing specific legal exclusions that permit attenuated variants like the LVS to be manipulated in BSL-2 environments.

The utilization of these strains for research purposes is fundamentally hierarchical. While LVS serves as an accessible tool for screening basic molecular interactions, FSC200 represents a modern platform for the construction of safer, next-generation vaccines through defined deletions. Schu S4 remains essential in models intended to demonstrate the efficacy of novel antimicrobial agents or vaccines. The high pathogenicity of the FSC200 and Schu S4 strains may limit certain studies due to the high lethality observed in laboratory animal models. Conversely, the LVS induces a more attenuated disease progression in laboratory animals, rendering it more suitable for applications such as antibody isolation. Investigating the discrepancies between these lineages at the transcriptomic and proteomic levels currently allows for the identification of novel targets for therapeutic interventions, specifically those factors responsible for the suppression of the host immune response in wild-type strains.

## 4. Antigen Preparation

The isolation of antigenic fractions from *F. tularensis* represents a critical phase of immunological research, requiring a precise balance between the efficiency of cellular disintegration and the preservation of the native conformation of protein and carbohydrate epitopes. Due to its high virulence and status as a facultative intracellular pathogen, all initial procedural steps—including cultivation and biomass harvesting—must be conducted under BSL-2 (FSC200 and LVSs) or BSL-3 (Schu S4 strain) containment. The process begins with the preparation of a standardized bacterial suspension; upon reaching the late logarithmic growth phase, cells are harvested via centrifugation and subsequently washed with an appropriate isotonic buffer. The choice of buffer is paramount; phosphate-buffered saline (PBS) at pH 7.2–7.4 or Tris–HCl buffer with sodium chloride are most frequently utilized to ensure osmotic stability and stabilize membrane surface electrostatic interactions [102,103,104]. Protease inhibitors, such as PMSF or cocktails containing EDTA and benzamidine, are often added to these matrices to prevent the unwanted proteolytic degradation of protein antigens by endogenous enzymes released during lysis. Following antigen preparation, safety verification via a sterility check of an aliquot is mandatory for infectious strains before further handling.

### 4.1. Disintegration of Frozen Cellular Suspensions by Pressure

One of the most effective methods for obtaining a comprehensive set of antigens, including those sequestered in the cytosol, is mechanical disintegration via high-pressure homogenization, commonly known as the French press technique [105,106,107]. This process utilizes the principle of controlled extrusion of a bacterial suspension through a narrow valve under high pressure (depending on particular type of cell, valve diameter and device manufacturer, typically around 1000 psi and higher for *F. tularensis*). A specific modification for Francisella research involves the crushing of frozen bacterial suspensions; the biomass is first flash-frozen to cryogenic temperatures (using liquid nitrogen or deep-freeze units) and subsequently subjected to mechanical pressure in a semi-solid state. In this context, ice crystals function as an abrasive medium that mechanically disrupts the highly resilient cell wall and capsular structures of the microorganism. The primary advantage of this method is the minimization of thermal stress, as frictional heat is immediately offset by the low temperature of the sample, which is vital for preserving thermolabile proteins such as components of the Type VI Secretion System (T6SS).

### 4.2. Freeze–Thaw Cycles and Thermal Disintegration

Another sophisticated approach for antigen release is the freeze–thaw lysis method [108,109], which has been successfully described for *F. tularensis* [110]. This procedure involves the successive exposure of the suspension to temperatures of approximately −196 °C (in liquid nitrogen) followed by rapid thawing in a 37 °C water bath. During freezing, the formation of intracellular ice crystals mechanically disrupts the integrity of both the cytoplasmic membrane and the peptidoglycan layer. Although this method is highly gentle regarding antigen structure and does not require expensive instrumentation, achieving high yields with *F. tularensis* often necessitates 5 to 10 cycles, which may induce the aggregation of certain hydrophobic proteins. To enhance efficiency, this protocol is occasionally combined with mild enzymatic treatment, such as the addition of lysozyme, which specifically cleaves the glycosidic bonds in peptidoglycan to facilitate subsequent mechanical lysis.

The efficiency of freeze–thaw cycles for *F. tularensis* can be significantly enhanced by modulating the composition of the lysis buffer; specifically, the addition of mild chaotropic agents or low-concentration detergents facilitates the destabilization of hydrophobic interactions within the cell wall. This synergistic effect allows for a reduction in the total number of cycles required, thereby minimizing the risk of protein aggregation and ensuring a higher yield of native membrane complexes.

Thermal disintegration (heat inactivation) constitutes a specific method of antigen preparation in which the bacterial suspension is exposed to temperatures ranging from 56 °C to 100 °C for a defined duration. This process induces the irreversible denaturation of proteins and disrupts the integrity of the cellular envelopes, thereby releasing thermostable components, most notably LPS and heat-shock proteins. Although this method results in the loss of tertiary structure for thermolabile protein epitopes, the resulting “heat-killed” antigen provides a high safety profile for subsequent handling and is frequently utilized as a standard reagent in agglutination assays or for primary immunization during the generation of antisera.

### 4.3. Sonication

In addition to the aforementioned mechanical procedures, sonication, the application of ultrasonic waves exceeding 20 kHz and energy above 1000 W, is commonly employed in laboratory practice [111]. Ultrasound induces cavitation within the suspension, the formation and subsequent collapse of microscopic vapor bubbles, which generate localized shock waves capable of shearing bacterial cells [112,113]. When sonicating *F. tularensis*, it is essential to utilize a pulsed mode and maintain constant cooling in an ice bath to prevent the denaturation of antigens by localized overheating. This method is excellent for DNA fragmentation, which reduces the viscosity of the resulting lysate and facilitates downstream purification steps such as affinity chromatography or ultrafiltration. If the objective is the specific isolation of outer membrane antigens, such as the FopA or Tul4 proteins, differential extraction using detergents like sarcosyl or Triton X-100 is employed to selectively solubilize specific membrane structures.

### 4.4. Secondary Processing and Stabilization of Antigens

The resulting crude extract, containing a mixture of proteins, LPS, and nucleic acids, must be cleared of cellular debris via centrifugation. Further processing involves the isolation of specific fractions through ultracentrifugation or chromatography [114,115,116,117]. Antigenic preparations in a liquid state are highly unstable and prone to rapid degradation. For long-term preservation of both cells and antigens, deep-freezing to temperatures of −80 °C or lower is the preferred method [118,119,120]. To improve stability, cryoprotectants—most commonly glycerol at concentrations of 10–20% or sucrose—are added to the suspension to prevent the formation of large ice crystals and protect the tertiary structure of proteins.

If the antigens must be transportable or remain stable at higher temperatures for extended periods, lyophilization (freeze-drying) is performed [121]. During this process, water is removed from the frozen sample via sublimation under vacuum, resulting in a stable “cake” (lyophilizate) that retains its original biological and immunological activity upon reconstitution in buffer. For specific immunological assays, such as ELISA or T-lymphocyte stimulation studies, it is necessary to define the purity and concentration of the acquired antigens. This is achieved through a combination of dialysis, which removes low-molecular-weight impurities and residual detergents, and subsequent protein quantification using standard colorimetric assays (e.g., Bradford or BCA assays). In the case of *F. tularensis*, it is also crucial to monitor the presence of LPS, which possesses a unique structure in this species (binding to TLR2 rather than TLR4); its concentration in the antigenic preparation can significantly influence the results of immunostimulatory tests. A correctly prepared and preserved antigen serves as the foundational reagent for diagnostic kit development, the validation of novel vaccine candidates, and a deeper understanding of the host–pathogen interface.

## 5. Anti-Tularemia Antibody Production: Antigen Selection and Characterization for Immunization

The selection of an appropriate antigenic determinant is the critical factor for the successful generation of antibodies characterized by high affinity and specificity. In the study of *F. tularensis*, research focuses primarily on surface structures that are directly exposed to the host’s immune system.

The LPS of *F. tularensis* is unique due to its structural configuration and low endotoxic activity, which results from the specific composition of its lipid A moiety (specifically, its tetra-acylated and dephosphorylated state) [122,123]. For antibody production, the O-antigen (O-polysaccharide) is also highly significant as it exhibits high immunogenicity [124,125,126]. Antibodies directed against the O-antigen are frequently protective and highly species-specific, making them invaluable for rapid diagnostics. During preparation, however, it must be noted that the isolation of pure LPS necessitates modified extraction protocols—such as the hot phenol–water extraction method—to prevent the degradation of the carbohydrate chains.

Outer membrane proteins (OMPs) serve as specific markers suitable for antibody production when specificity against surface-exposed proteins is required. These OMPs represent ideal targets for the development of monoclonal antibodies. Among the most extensively characterized are the following: FopA (*Francisella* outer membrane protein A): a protein highly conserved across all subspecies, frequently utilized as a diagnostic marker [127,128,129]; Tul4 (LpnA): an immunoreactive lipoprotein known to induce a robust humoral immune response [130,131]; and IglC: a protein associated with the Type VI Secretion System (T6SS), which is essential for the pathogen’s escape from the phagosome [132,133].

For advanced applications, recombinant antigens produced in heterologous expression systems (most commonly *Escherichia coli*) are utilized. In these instances, a critical evaluation of the absence of post-translational modifications (particularly glycosylation) is necessary, as these modifications may be essential for correct antibody recognition of the native protein.

The humoral immune response to *F. tularensis* is dominated by antibodies directed at the LPS O-antigen, a structure that remains chemically and immunologically identical across the highly virulent subsp. *tularensis* and the moderately virulent subsp. *holarctica* [134]. Because this primary immunodominant marker is conserved, standard serological assays generally cannot distinguish between exposures to different subspecies based on anti-LPS titers alone. However, the host’s overall immune profile can differ, as infections by subsp. *tularemia* often trigger a more suppressed early innate response followed by a more aggressive systemic inflammation compared to subsp. *holarctica*. While major outer membrane proteins like FopA and Tul4 are also highly conserved and elicit cross-reactive antibodies, recent diagnostic advancements have identified rarer, subspecies-specific protein markers [135]. For instance, specific antibodies targeting the PilT protein or FTT0571 can uniquely identify subspecies *tularensis*, whereas markers like FTL0187 are used to specifically recognize subspecies *holarctica* in specialized Western blot or ELISA formats [136,137].

### 5.1. Production of Polyclonal Antibodies

Polyclonal antibodies represent a heterogeneous mixture of immunoglobulins that recognize multiple epitopes on a single antigen. Their primary advantages include the ability to form stable immune complexes and enhanced sensitivity in assays such as ELISA or Western blotting.

The most common host organisms to produce polyclonal antibodies are pigs, rabbits, and goats, while laboratory mice and rats are predominantly utilized for experimental-scale preparation [138,139]. In experimental settings where smaller volumes of antiserum are required, rodent models are advantageous. The choice of host depends on the required volume of antiserum. Immunization typically involves a primary dose of the antigen emulsified with Complete Freund’s Adjuvant (CFA), followed by several booster doses administered with Incomplete Freund’s Adjuvant (IFA) [140,141]. In the specific case of *F. tularensis*, inactivated whole-cell lysates are frequently employed as antigens. This approach yields broad-spectrum antisera; however, such sera often necessitate subsequent adsorption against cross-reacting microorganisms to enhance specificity [142].

The production of antibodies via in vivo induction through the experimental infection of a model organism represents a specialized approach. Unlike immunization with isolated antigens, this method allows the host’s immune system to respond to the full spectrum of native epitopes in their natural conformation and quantitative distribution. This process simulates natural pathogenesis, leading to the production of convalescent sera characterized by high affinity, which often exhibit superior neutralizing and opsonizing activity.

For the generation of polyclonal antibodies via this route, pure antigens or mixtures are replaced by experimental infection followed by seroconversion. Laboratory rats (e.g., Fischer 344), rabbits, and mice (e.g., the BALB/c strain) are the most frequently utilized models. The process initiates with the application of a sublethal dose (typically expressed as a fraction of the LD50) of an attenuated strain, most commonly LVS, which minimizes the risk of high lethality. The infection is administered subcutaneously or intranasally to stimulate both systemic and mucosal immune responses.

Following the resolution of the acute phase of infection, a specific humoral response develops. To achieve maximal titers of IgG isotypes, hyperimmunization (a “booster”) may be performed after a defined interval (typically 21–28 days), during which the animal is challenged with inactivated antigen or cell wall fragments. This step induces the proliferation of memory B-lymphocytes and subsequent affinity maturation of the antibodies within the germinal centers of the lymph nodes.

### 5.2. Generation of Monoclonal Antibodies

Monoclonal antibodies provide the advantage of unlimited reproducibility and defined specificity toward a single epitope [143,144]. They are generated using hybridoma technology [145,146,147]. The standard procedure involves the fusion of B-lymphocytes harvested from immunized mice (typically the BALB/c strain) with myeloma cells (e.g., the Sp2/0 line). Following selection in HAT medium (Hypoxanthine–Aminopterin–Thymidine), the process enters the critical screening phase. For *F. tularensis*, it is essential to evaluate reactivity not only against the primary immunogen but also against closely related species to exclude non-specific clones.

To eliminate the requirement for laboratory animals and accelerate development timelines, libraries of scFv fragments (single-chain variable fragments) are increasingly utilized. This technology enables the in vitro selection of antibodies against highly toxic or weakly immunogenic components of *Francisella*. The resulting recombinant fragments can subsequently be “humanized” or modified to enhance binding affinity through techniques such as error-prone PCR or DNA shuffling.

### 5.3. Antibodies Isolation and Purification of Immunoglobulins

The purity of the acquired antibodies is fundamental to their stability and functionality within bioanalytical systems. The purification process typically proceeds through several stages. Precipitation and dialysis represent effective methods for purifying antibodies obtained under both experimental and biotechnological production conditions. The initial step for antisera or culture media often involves “salting out” with ammonium sulfate, typically at a saturation of 30% to 50% [148,149]. This step facilitates the removal of the majority of albumin. Subsequent dialysis against a physiological buffer (e.g., PBS, pH 7.4) is performed to remove residual salts. Affinity chromatography is the primary tool for achieving high immunoglobulin purity. For maximal refinement, liquid chromatography is employed: Protein A/G Chromatography: This technique exploits the high affinity of bacterial proteins for the Fc fragments of IgG. It is a universal method for the total isolation of the IgG fraction [150,151,152]. Antigen-Specific Purification: The target antigen is immobilized onto a solid support (e.g., Sepharose). This approach allows for the selective isolation of only those antibodies from a polyclonal antiserum that directly recognizes the specific antigen. Elution is achieved by altering the pH (e.g., using 0.1 M glycine–HCl, pH 2.5–2.8), followed by immediate neutralization [153,154,155].

### 5.4. Specificity and Cross-Reactivity

The primary challenge regarding antibodies directed against *F. tularensis* is cross-reactivity with the genera Brucella and Yersinia, as well as other species within the genus *Francisella* [5,156,157,158,159]. Specificity is fundamentally determined by the molecular structure of the epitope. While antibodies targeting the O-antigen demonstrate high specificity for the *tularensis* and *holarctica* subspecies, those directed against highly conserved proteins—such as the chaperonin GroEL—exhibit broad cross-reactivity across a wide range of Gram-negative bacteria. Consequently, differential diagnostics necessitate epitope mapping and the utilization of antibody “cocktails” (combinations of several monoclonal antibodies) targeting distinct surface domains to ensure diagnostic accuracy.

### 5.5. Storage and Stabilization Methodologies

As proteins, antibodies are susceptible to denaturation, aggregation, and proteolytic degradation; therefore, rigorous storage protocols are essential to preserve their antigen-binding capacity [160,161,162]. For short-term storage (extending over several weeks), a temperature of 4 °C is appropriate, provided that bacteriostatic agents such as sodium azide are added at concentrations of 0.02% to 0.05% to prevent microbial contamination. For long-term preservation, temperatures ranging from −20 °C to −80 °C are required. It is imperative to avoid repeated freeze–thaw cycles, as the resulting mechanical stress leads to the fragmentation of the IgG molecules. Recommended practices include aliquoting the samples and incorporating cryoprotectants, such as glycerol (10–50%) or ethylene glycol.

Lyophilization (freeze-drying) represents the most stable form of preservation [163,164]. This process requires the use of lyoprotectants (e.g., sucrose or trehalose), which substitute for the protein’s hydration shell and prevent structural collapse during the sublimation of water. Lyophilized antibodies can be maintained at room temperature for several years, a characteristic that is critical for the deployment of field-ready diagnostic kits.

## 6. Standard Immunoassays for *F. tularensis* Detection and Tularemia Diagnosis

In the clinical diagnostic hierarchy for *F. tularensis*, traditional microbiological techniques often encounter significant practical hurdles. While gold-standard cultivation remains the definitive proof of infection, the fastidious nature of this Gram-negative coccobacillus requires enriched media, such as cysteine heart agar, and often takes several days to yield visible colonies. Microscopy, typically utilizing Gram or Giemsa stains, frequently proves insufficient due to the organism’s minute size and poor staining characteristics. Some devices, such as the VITEK 2 Gram-negative identification card, can serve for preliminary screening [165]. Furthermore, while nucleic acid amplification tests like polymerase chain reaction (PCR) or Lop-Mediated Isothermal Amplification (LAMP) offer exceptional sensitivity and rapid results, they require specialized equipment and are primarily effective during the transient bacteremia phase [166,167,168,169,170,171]. Consequently, immunoassays serve as the indispensable backbone of tularemia diagnostics. These assays are categorized into direct methods, which utilize labeled antibodies to sequester and identify bacterial antigens (primarily lipopolysaccharide), and indirect serological methods, which quantify the host’s humoral response. The following sections focus exclusively on the immunological principles and platform-specific characteristics of these diagnostic tools. A survey of the discussed immunoassays is depicted as Table 3.

The fundamental principle of these immunoassays rests on the biochemical specificity of the paratope–epitope interaction. In a diagnostic context, this involves the formation of an immune complex between *F. tularensis* antigens, most commonly the highly immunogenic O-antigen of LPS, and specific immunoglobulins. Direct assays, such as lateral flow assays, function by “sandwiching” the target antigen between a stationary capture antibody and a mobile, labeled detection antibody, producing a localized signal. Indirect assays, such as ELISA or agglutination, measure the presence of host-derived IgM, IgG, or IgA. These interactions are visualized through various signal transduction mechanisms: enzymatic conversion of substrates into colored products in ELISA, the physical lattice formation and precipitation of insoluble complexes in agglutination, or the enzymatic breakdown of chemiluminescent reagents in more sensitive formats.

Pathogen identification frequently utilizes lateral flow assays (LFAs), which operate on a sandwich-based immunochromatographic principle. As the specimen migrates through a functionalized membrane, target cells are bound by stationary antibodies, while secondary labeled reagents provide a discernible signal. These platforms offer unrivaled speed for onsite diagnostics, though their higher detection limits mean they cannot exclude infection with the same certainty as nucleic acid tests. While commercial versions are widely deployed, academic innovation persists in enhancing the sensitivity of these accessible diagnostic tools. Commercially available LFAs are applicable for routine detections of *F. tularensis* [172,173]. Development of LFAs is nevertheless still ongoing and improved tests became introduced [174].

For retrospective or late-stage diagnosis, serological assays that detect specific IgM and IgG antibodies are standard, but it is also suitable for detection of antigen [174,175,176,177,178]. As a standard clinical tool, ELISA utilizes microtiter plates coated with LPS or inactivated whole cells to sequester host antibodies from patient serum. These bound markers are subsequently identified by enzyme-linked conjugates to produce a measurable signal. The platform’s primary strength lies in its high-throughput efficiency and its capacity to provide semi-quantitative insights into the progression of the host’s immune response. Despite its utility, the assay is plagued by the “diagnostic window,” as antibodies typically do not reach detectable levels until the second week of illness.

Agglutination-based assays, including the Microagglutination Test (MAT) and Tube Agglutination Test (TAT), represent some of the oldest yet most resilient methodologies in tularemia diagnostics [179,180,181,182,183]. The principle involves the visible clumping of bacterial antigens when cross-linked by bivalent antibodies in the patient’s serum. MAT is particularly favored in reference laboratories because it requires minimal specialized instrumentation and provides a clear titer of the total antibody response. Nevertheless, these tests are subjective and can be influenced by the “prozone effect,” where an excess of antibodies prevents the formation of a visible lattice, leading to false-negative interpretations.

Confirmatory testing often necessitates the use of immunoblotting or dot–blot assays [184,185,186]. In a Western blot, *F. tularensis* proteins and LPS are separated by electrophoresis and transferred to a membrane. This allows for the visualization of the characteristic “LPS ladder” pattern, which is highly specific to the genus. By identifying the exact molecular weight of the antigens being targeted by the host’s immune system, clinicians can distinguish a true *F. tularensis* infection from cross-reacting antibodies generated against Brucella abortus or *Yersinia enterocolitica*. The structural homology of the O-antigen between these species is a significant source of diagnostic interference, often requiring differential testing to ensure accuracy.

The interpretation of these immunoassays must always be contextualized within the patient’s clinical history and the local epidemiology. A significant limitation across all serological platforms is the persistence of antibodies; IgG can remain detectable for years following recovery, making it difficult to distinguish between an acute infection and past exposure. Furthermore, early administration of aminoglycosides or fluoroquinolones can suppress the magnitude of the antibody response, potentially leading to false-negative results in the acute phase. Therefore, a fourfold rise in titer between acute and convalescent sera remains the definitive serological evidence for an active case of tularemia.

Addressing the inherent limitations of standard immunoassays, such as the protracted “diagnostic window” for antibody production and the suboptimal sensitivity of standalone direct antigen tests, requires a shift toward integrated multiplexing platforms. A significant diagnostic gap exists in the current reliance on separate assays to detect either the microbial presence or the host response. By developing dual-detection systems that simultaneously target the highly immunogenic O-antigen of the *F. tularensis* LPS and host-derived IgM/IgG, clinicians could theoretically close the early-stage window when bacterial loads are present, but antibodies remain undetectable. This approach mirrors the success of fourth-generation HIV diagnostics, which combine p24 antigen detection with antibody screening to provide more reliable results during the acute phase of infection.

Implementing such a hybrid format for *F. tularensis* would not only improve early-stage triage but also provide crucial context for interpreting results in areas where antibodies persist for years post exposure. By quantifying the pathogen load alongside the kinetics of the immune response in a single diagnostic window, these multiplexed tools could mitigate the diagnostic interference typically encountered in serological platforms.

## 7. Biosensors and Biosensors Like Devices

Advanced biosensors integrate antibody-based recognition with physicochemical transducers to convert binding events into measurable optical, electrical, or mechanical signals. Designed for biodefense and point-of-care applications, these devices bypass laboratory delays by enabling real-time detection of *F. tularensis* cells or LPS within complex clinical and environmental matrices. Their architecture typically involves immobilizing antibodies on functionalized surfaces where molecular interactions are immediately transformed into quantifiable data.

### 7.1. The Raptor Platform

The Raptor platform (by Research International Inc., Monroe, Washington, DC, USA) represents a premier example of an evanescent wave biosensor for *F. tularensis* and other biological warfare agents that has been successfully transitioned from laboratory development into practical, field-ready application [187,188,189,190,191]. Designed as a ruggedized, portable analytical system, the device bridges the gap between high-sensitivity benchtop fluoroimmunoassays and the necessity for rapid, on-site biohazard screening. Its deployment in both military and civilian defense sectors underscores its reliability as an automated tool for monitoring biological threats in real-time. The Raptor is engineered for portability and durability in demanding environments, featuring a compact “briefcase”-style form factor. It typically weighs approximately 5.6 kg (12 lbs), with dimensions of roughly 27.4 cm in width, 18.6 cm in depth, and 27.4 cm in height, making it easily transportable by a single operator. The external housing is constructed to withstand physical shocks and environmental exposure, protecting the internal precision optics and fluidic modules. The user interface consists of an integrated liquid crystal display (LCD) and a tactile keypad, allowing standalone operation without the requirement for an external computer, although data can be exported via RS-232 or USB interfaces for deeper kinetic analysis. The device operates on an internal battery for field use but is also compatible with standard AC power sources. While the detection of *F. tularensis* is a primary application, the Raptor is a versatile multi-analyte workstation. It is designed to identify a broad spectrum of biological agents, ranging from bacterial cells and spores to viral particles and protein toxins. In addition to *Francisella*, the system is frequently configured to detect *Bacillus anthracis* (anthrax) spores, *Y. pestis* (plague), and various *Brucella* species. Beyond vegetative cells, it is highly effective at quantifying potent toxins such as ricin, staphylococcal enterotoxin B (SEB), and *Botulinum* neurotoxins. This multiplexing capability is facilitated by the four-channel handheld test design, where different fiber optic probes can be functionalized with specific antibodies for a customized “threat panel” tailored to the specific geographical or tactical context.

**Figure 2:**
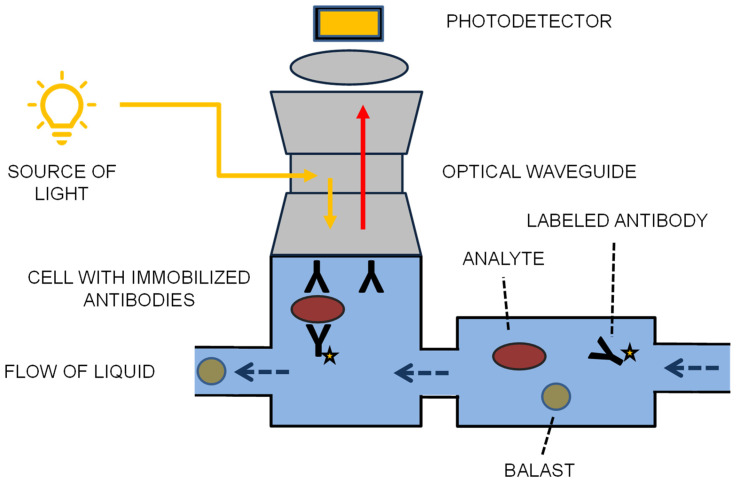
The principle of the Raptor device.

The operational cycle of the Raptor follows a sophisticated automated sequence within its specialized fluidic channels. In the initial phase, a sample containing the target analyte is aspirated into the disposable handheld test, where specific capture antibodies against *F. tularensis* are already immobilized on the surface of the polystyrene waveguides. During the subsequent binding step, the bacterial cells or LPS antigens are sequestered from the sample matrix onto the fiber surface. In the third phase, the system introduces a tracer antibody conjugated with a near-infrared fluorophore, specifically Cyanine 5 (Cy5) or Alexa Fluor 647, which recognizes a different epitope on the captured pathogen, effectively forming a molecular “sandwich.” Signal transduction occurs when a 635 nm diode laser (5 mW) light is coupled into the fiber, undergoing total internal reflection. This creates an evanescent field that penetrates only a small fraction of a micron into the surrounding fluid, typically between 100 and 200 nm. Consequently, only the fluorophores bound in the immediate vicinity of the fiber surface are excited, emitting a signal at approximately 670 nm that is proportional to the concentration of the *F. tularensis* load. This specific excitation geometry allows the device to operate with high precision even in turbid samples, as it inherently ignores fluorescence from unbound labels in the bulk solution. The principle of the assay is graphically summarized in Figure 2. The Raptor provides a rapid diagnostic window, yielding results in as little as 3 min for high-concentration samples, with a standard protocol typically lasting 5 to 15 min. For *F. tularensis*, the device achieves a limit of detection (LOD) in the range of 5 × 104 CFU/mL. Toxins were analyzed with LODs between 1 ng/mL (staphylococcal enterotoxin B, for instance) and 10 ng/mL (ricin). Its ability to process samples like whole blood or environmental slurries without extensive pre-filtration is a major logistical advantage. The system is designed for high efficiency; if a sample is negative, the probes can be automatically rinsed and reused for up to 40 subsequent assays before the handheld test must be replaced. Basic specifications of Raptor devices are summarized in Table 4.

The integration of automated rinsing cycles further enhances the device’s utility, allowing the same optical handheld test to be reused for continuous monitoring until a positive detection event occurs. This feature minimizes the cost per test and the volume of hazardous waste generated during long-term surveillance operations.

### 7.2. BioHawk LF, AnCam 6100 and QuikTest HHA

The BioHawk LF (Research International Inc., Monroe, Washington, DC, USA) is a high-level biosensor system that integrates large-volume aerosol collection with automated identification. It serves as a comprehensive platform designed for continuous, unattended monitoring of biological threats. The device utilizes a wetted-wall cyclonic sampler with a high airflow rate of 325 L per minute, which effectively concentrates airborne particles into a small 4.5 mL liquid volume. This collector is paired with an onboard ultraviolet biofluorescence detector that monitors the environment for sudden increases in biological particulate matter.

When a trigger event occurs, the system initiates an automated assay protocol using an eight-channel LFA. BioHawk LF employs a sophisticated machine-vision system to examine the development of the test strips, providing results within 10 to 25 min. Structurally, the device is built for rugged use, with dry weight specifications of approximately 13 kg and physical dimensions of 47 cm × 25 cm × 36.5 cm. It operates on 28 VDC or standard AC mains. A critical feature of this system is its “auto-flush” protocol, which decontaminates the internal fluidics between assays, allowing for long-term deployment without manual intervention. While *F. tularensis* is a primary target, the system is designed to detect a broad spectrum of agents, including *Bacillus anthracis* and various protein toxins.

The AnCam 6100 (Research International Inc.) is a compact, portable multi-analyte reader that acts as an objective bridge between manual tests and laboratory-grade data. Weighing only 1.28 kg (2.8 lbs) and measuring 20.5 cm × 12.1 cm × 10.9 cm, it is specifically designed to eliminate the subjective nature of human eye interpretation. The device utilizes high-resolution machine vision and proprietary signal processing algorithms to analyze the test strips under controlled internal lighting.

For the detection of *F. tularensis*, the AnCam 6100 offers a significant advantage in speed. While standard LFA tickets require a 15 min incubation, the AnCam’s algorithms can often detect a strong positive for *F. tularensis* as early as 5 min into the process. The device provides real-time estimates as the assay progresses and archives the results with GPS location stamps. Data can be transmitted via cellular or WiFi networks, and the unit is powered by an internal lithium-ion battery, making it a vital tool for decentralized field diagnostics. It is compatible with various handheld test formats, including those with one, five, or eight analyte channels.

The QuikTest HHA (Hand-Held Assay by Research International Inc. Monroe, Washington, DC, USA) is the primary immunochromatographic sensing consumable utilized by both BioHawk LF and AnCam 6100. These assays utilize a sandwich-format principle involving colloidal gold-labeled antibodies that bind to target antigens like the *F. tularensis* lipopolysaccharide. The resulting complex migrates to a capture zone to form a visible reddish-brown line.

Analytical specifications for the QuikTest HHA indicate a LOD for bacterial agents like *F. tularensis* at approximately 100,000 CFU/mL, while protein toxins can be detected at levels between 1 and 10 ppb. The assays are engineered for high stability, with a shelf life of 12 to 18 months when stored at room temperature. Beyond *F. tularensis*, these tickets are validated for agents such as *Brucella* spp., ricin, and staphylococcal enterotoxin B. The credit-card-sized tickets are designed for ease of use, weighing less than 50 g and requiring no external power for the biochemical reaction to occur. The comparative specifications of BioHawsk LF, AnCam 6100, and QuikTest HHA are written in Table 5.

The combination of three components, automated sampling, objective interpretation, and stable immunochromatographic sensing, provides a robust framework for the rapid identification of *F. tularensis* in high-risk environments.

### 7.3. Comparison of the Commercial Biosensors Like Devices

When evaluating the practical deployment of automated biodefense systems for *F. tularensis*, the selection between the Raptor and BioHawk LF platforms depends on whether the operational requirement prioritizes portability or autonomous environmental surveillance. The Raptor system offers a highly portable “briefcase” form factor, weighing only 5.6 kg, which allows a single operator to conduct high-sensitivity evanescent wave fluoroimmunoassays in the field. In contrast, the BioHawk LF is a significantly heavier unit at 13 kg, designed for fixed-position or vehicle-mounted use where its integrated 325 L/min cyclonic air sampler can continuously monitor the environment. While the Raptor requires manual sample aspiration into its four-channel test plates, the BioHawk LF provides a fully automated workflow, triggered by an internal ultraviolet biofluorescence detector that initiates eight-channel lateral flow assays upon detecting spikes in atmospheric particulate matter.

From a cost-effectiveness and field-validation perspective, both systems utilize sophisticated methods to minimize the logistical burden of long-term monitoring. The Raptor platform is particularly efficient for screening multiple negative samples, as its fiber-optic probes can be automatically rinsed and reused for up to 40 consecutive assays, significantly reducing the cost per test and the accumulation of hazardous waste. The BioHawk LF similarly employs an “auto-flush” protocol to decontaminate its internal fluidics, enabling unattended long-term deployment. Regarding analytical performance, the Raptor achieves a LOD for *F. tularensis* at 5 × 10^4^ CFU/mL, slightly outperforming the QuikTest HHA strips used by the BioHawk, which typically detect the pathogen at concentrations around 10^5^ CFU/mL. Ultimately, the Raptor is best suited for targeted, rapid diagnostics in decentralized settings, while the BioHawk LF excels in automated, continuous surveillance of high-risk public or military environments.

### 7.4. Developed Biosensors and Biosensors-like Devices

Building upon the commercial success of platforms like the Raptor, BioHawk, and various handheld assays, recent academic advancements have introduced a diverse array of experimental biosensors that push the boundaries of sensitivity and speed. These laboratory-developed systems utilize novel transducers and nanomaterial-enhanced recognition layers to address the specific challenges of *F. tularensis* detection in clinical and environmental settings. A critical advantage that distinguishes these advanced biosensing platforms from traditional laboratory techniques, such as the ELISA, is the dramatic reduction in operational turnaround time. While conventional ELISA protocols typically necessitate several hours due to protracted incubation periods and multiple sequential washing steps, modern biosensors facilitate rapid diagnostics, often concluding an entire analytical cycle in under thirty minutes. Furthermore, the integration of high-performance nanomaterials and sensitive transduction surfaces frequently allows these devices to surpass the detection limits of standard methods. By achieving superior sensitivity and near-real-time results, these biosensors provide a decisive edge in scenarios requiring immediate clinical intervention or urgent environmental screening for *F. tularensis*. A survey of such devices is given in Table 6.

The research presented in the study by Sphehar-Deleze et al. details the implementation of an electrochemiluminescence immunosensor specifically engineered for the high-sensitivity detection of *F. tularensis* [192]. The platform utilizes a sandwich immunoassay format localized on a screen-printed gold electrode array integrated into a custom-built fluidic chip. The assay employs ruthenium-based labels, specifically Ru(bpy)32+-encapsulated silicate nanoparticles conjugated to secondary antibodies, to generate the luminescent signal upon electrochemical excitation. A notable aspect of this study is the comparative evaluation of whole antibodies versus antibody F(ab) fragments as capture biomolecules, with the latter providing superior orientation and binding efficiency. Analytically, the ECL sensor demonstrates exceptional performance, reaching a LOD of 70 bacteria/mL when using whole antibodies and an even more impressive 45 bacteria/mL with F(ab) fragments. For purified LPS, the LOD was established at 0.4 ng/mL. The device is housed in a “black box” system that integrates the necessary optics, fluidics, and electronics for rapid analysis. By utilizing nanoparticle-encapsulated labels, the system achieves significant signal amplification, allowing for the detection of *F. tularensis* at concentrations several orders of magnitude lower than traditional LFA or standard electrochemical assays. The exact time per one assay cycle is not mentioned, but the sum of the incubation steps last 25 min, while the overall time per one assay cycle can be around 30 min.

The research conducted by Jang and coworkers details the engineering of hierarchical dual-mode nanoprobes designed for the simultaneous Surface-Enhanced Raman Scattering (SERS) and fluorescence detection of *F. tularensis* [193]. These complex nanostructures consist of metallic nanoparticle clusters integrated with Raman-active reporters and fluorescent dyes, all encapsulated within chemically stabilized polymeric nanoparticles via electrohydrodynamic jetting. The assay utilizes a sandwich-type immunocomplex formation where monoclonal antibodies against *F. tularensis* are conjugated to both the dual nanoprobes and magnetic beads. The magnetic beads facilitate rapid pathogen isolation and concentration, while the dual nanoprobes provide two distinct signals: a SERS signal for precise quantification and a fluorescence signal for rapid bioimaging at excitation wavelengths of 514 nm and 633 nm. Analytically, the SERS platform achieves a LOD of less than 10^2^ cells/mL. The system demonstrated a robust linear correlation between Raman signal intensity and pathogen concentration across a dynamic range of 10^2^ to 10^6^ cells/mL. This dual-modal approach is particularly significant for multiplexed applications, as demonstrated by the simultaneous detection of Escherichia coli and *F. tularensis* without signal overlap. By combining the high sensitivity and fingerprinting capabilities of SERS with the rapid imaging speed of fluorescence, the probes offer a powerful tool for both qualitative visualization and quantitative assessment of bacterial pathogens.

The study conducted by Nualnoi and colleagues explores the optimization of a sandwich-format antigen capture ELISA for the detection of *F. tularensis* LPS [194]. The primary focus of the research was the systematic enhancement of assay sensitivity through immunoglobulin subclass switching of the monoclonal antibody 1A4. Initially, an IgG3-based assay exhibited suboptimal performance due to high background signaling, which was subsequently attributed to the self-association tendencies of the IgG3 constant region. By transitioning to IgG1 and IgG2b variants within the same subclass-switch family, the researchers successfully mitigated these non-specific interactions. Analytical evaluation revealed that while Surface Plasmon Resonance (SPR) indicated a decrease in functional affinity following the switch from IgG3, the overall assay sensitivity improved significantly due to the drastic reduction in background noise. The optimized IgG2b-based ELISA achieved a LOD for purified LPS of approximately 0.6 ng/mL, while the LOD for whole *F. tularensis* (LVS) was established around 2.5 × 10^4^ CFU/mL. This research underscores the critical impact of antibody isotype on the signal-to-noise ratio in diagnostic platforms targeting the surface-expressed O-antigen of the pathogen. The time per one assay is not mentioned in the study but it can be estimated between 10 and 20 min depending on exact configuration of the assay and particular incubation times.

The research presented by Devadhasan and colleagues details the development of VeriFAST, an integrated vertical flow immunoassay platform designed for the rapid multiplexed detection of Tier 1 biothreat agents [195]. Unlike traditional LFA systems, the VeriFAST architecture utilizes a vertical fluidic path through a functionalized nitrocellulose membrane, which is coupled with automated fluid handling and smartphone-based digital image processing. The assay employs a gold nanoparticle-based sandwich format to simultaneously target multiple antigens, including the LPS of *F. tularensis*, alongside antigens of *Y. pestis* and *Burkholderia pseudomallei*. Analytical characterization of the system demonstrates high sensitivity across diverse and complex matrices, including serum, urine, and soil extracts, with a total operational time of less than 30 min per one assay cycle. Specifically, the platform achieved a LOD for *Y. pestis* and *B. pseudomallei* antigens between 0.0125 and 0.625 ng/mL while the assay of *F. tularensis* LPS was not as effective due to high matrix signal. Stability assessments indicated that while detection antibodies and buffers remain viable for 12 months at ambient temperatures, capture antibodies are more sensitive to environmental degradation, showing signal drift after 3 months. This system addresses the need for objective, field-portable diagnostics by combining automated sample processing with mobile analytics to ensure reproducible results in resource-limited settings.

## 8. Conclusions

The effective management of *F. tularensis* infection and the mitigation of its potential as a biological weapon are inextricably linked to the availability of rapid, sensitive, and field-deployable diagnostic systems. As detailed throughout this manuscript, the evolution of immunoassays has moved beyond the constraints of traditional laboratory-based cultivation and standard serology. The integration of advanced biotechnological approaches, such as the production of high-affinity monoclonal antibodies and the use of recombinant antigens, has laid the foundation for a new generation of diagnostic tools.

Biosensors and various biosensor-like devices, including vertical flow immunoassays and handheld optical platforms, represent the current frontier in this field. These technologies are particularly vital for early diagnosis, where the prompt initiation of specific antimicrobial therapy can drastically reduce mortality rates. The ability to perform high-sensitivity detection in point-of-care settings or directly in the field, without the need for complex laboratory infrastructure, is a critical advantage in both public health and biodefense contexts.

Furthermore, the implementation of automated sampling and smartphone-based digital processing has enhanced the objectivity and accessibility of these tests. While challenges regarding cross-reactivity with related genera persist, the development of multi-target antibody cocktails and dual-mode detection systems continues to improve diagnostic specificity. In summary, the continued refinement of biosensor technology remains the most promising pathway toward the early detection and effective control of this significant infectious agent, ensuring a more robust response to both natural and anthropogenic tularemia outbreaks.

## Figures and Tables

**Table 1:** The subspecies of *Francisella tularensis*.

Subspecies	Common Type	Geographic Distribution	Virulence Level	Primary Ecology and Impact	References
subsp. *tularensis*	Type A	Predominantly North America	Extremely High	Highly lethal; requires BSL-3 containment; primary bioterrorism concern.	[21,22]
subsp. *holarctica*	Type B	Northern Hemisphere (Europe, Asia, North America)	Moderate to High	Wide zoonotic circulation; common cause of water-borne and vector-borne outbreaks.	[23,24,25]
subsp. *mediasiatica*	N/A	Central Asia	Moderate/Low	Ecological niche restricted to specific arid regions; clinical data in humans is limited.	[26,27,28]
subsp. *novicida* or *F. novicida* in some sources	N/A	Worldwide (rarely isolated)	Low (Opportunistic)	Non-pathogenic in immunocompetent humans; used as a primary laboratory surrogate model.	[29,30,31,32,33,34,35]

**Table 2:** Summary of primary discrepancies between the S4, FSC200, and LVSs.

Strain	Advantages for Research	Disadvantages for Research
Schu S4	Elucidation of maximum virulence; ideal for evaluating the definitive efficacy of vaccines and novel antibiotics within a model of the most severe infection course.	Extreme risk to personnel; requires costly BSL-3 containment; regulated as a potential bioterrorism agent; high mortality in animal models may preclude certain experiments (e.g., antibody production).
FSC200	Permissible under BSL-2 protocols; genetically stable Type B reference strain; suitable for reverse genetics and the development of targeted, defined attenuated mutants; faithfully models European tularemia.	High mortality in laboratory animal models may preclude specific experimental procedures (e.g., antibody production).
LVS	Permissible under BSL-2 protocols; widely accepted model for fundamental research into intracellular parasitism and general immunological principles; dose titration allows for host survival, facilitating applications such as antibody isolation.	Ambiguous mutational history and genetic instability (tendency for colony dissociation); demonstrates limited protective efficacy against Type A variants when used as a vaccine.

**Table 3:** Comparative analysis of immunoassay methodologies.

Assay Type	Target Analyte	Clinical Utility	Sensitivity	Specificity	Interference and Limitation Factors
ELISA	IgM/IgG/Antigen/Cells	Primary screening; surveillance	High	Moderate	Depends on used antibodies
MAT	Total Ab/Antigen/Cells	Standard reference; titration	Moderate	High	Prozone effect; subjective reading
LFA	LPS/Antigen/Cells	Point-of-care; rapid triage	Low/Moderate	High	Sample matrix effects; low titer
Dot-Blot	LPS/Antigen/Cells	Qualitative screening	Moderate	Moderate	Non-specific binding to membrane
Western Blot	Specific Proteins	Confirmatory diagnosis	High	Very High	Labor intensive; slow turnaround
Agglutination	Whole Cells, Serum, Plasma with Antibodies	Rapid field/lab screening	Moderate	Moderate	High bacterial load requirements
Immunoprecipitation	Soluble Antigens	Research/specific laboratories	Moderate	Moderate	Requires high-affinity antibodies

**Table 4:** Basic specifications of Raptor biosensor device.

Parameter	Specification
Detection Method	Evanescent wave fiber-optic fluoroimmunoassay
Target Analytes	*F. tularensis*, *B. anthracis*, *Y. pestis*, ricin, SEB, Botulinum
Excitation/Emission	635 nm/670 nm (near-infrared)
LOD	5 × 10^4^ CFU/mL for *F. tularensis*
Typical Assay Time	5 to 15 min
Weight	~5.6 kg (12 lbs)
Dimensions	~27.4 cm × 18.6 cm × 27.4 cm
Operating Power	Internal battery or AC power
Probe Capacity	4 independent channels per test plate
Probe Longevity	Up to 40 uses for negative samples

**Table 5:** Comparative specifications of BioHawsk LF, AnCam 6100, and QuikTest HHA.

Parameter	BioHawk LF	AnCam 6100	QuikTest HHA (Consumable)
Device Category	Autonomous detector/sampler	Portable machine-vision reader	LFA strip
Principal Analytes	*F. tularensis*, *B. anthracis*, *Y. pestis*, toxins	*F. tularensis*, *B. anthracis*, *Y. pestis*, toxins	*F. tularensis*, *B. anthracis*, *Y. pestis*, toxins
Primary Transduction	UV fluorescence + machine vision	Optical reflection/machine vision	Colorimetric (colloidal gold)
Detection Limit	100 to 100,000 CFU/mL (Bacteria)	N/A (Reader-dependent)	1 to 10 ppb (toxins); CFU/mL (bacteria)
Assay Time	10–25 min	Results in seconds (post incubation)	15 min (standard)
Weight	~13 kg (Dry)	1.28 kg (2.8 lbs)	<50 g
Physical Dimensions	47 cm × 25 cm × 36.5 cm	20.5 cm × 12.1 cm × 10.9 cm	Credit card-sized
Power Source	28 VDC/AC mains	Internal lithium-ion battery	None required
Multiplexing	Up to 8 simultaneous analytes depending on used test plate	1-, 5-, or 8-analyte test plate	1 to 8 analytes per handheld test plate

**Table 6:** Immunosensors for *F. tularensis* presented in scientific journal articles.

Principle	Analyte/Strain	LOD	Notable Feature	Reference
Electrochemiluminescence	*F. tularensis* LVS	45–70 cells/mL	use of F(ab) fragments and Ru nanoparticles	[192]
SERS and Fluorescence Detection	*F. tularensis* cells	10^2^ cells/mL	Immunocomplex formed on magnetic beads	[193]
Antigen Capture ELISA	*F. tularensis* LPS/LVS	0.6 ng/mL (LPS), 2.5 × 10^4^ CFU/mL (whole cells)	IgG subclass switching from IgG3 to IgG2b)	[194]
Vertical Flow Immunoassay	LPS from *F. tularensis*, alongside antigens of *Y. pestis* and *B. pseudomallei*	0.0125 and 0.625 ng/mL for *Y. pestis* and *B. pseudomallei*	Lack of sensitivity for LPS from *F. tularensis*	[195]

## Data Availability

All data are presented in this paper.
